# An investigation of nutrient-dependent mRNA translation in *Drosophila* larvae

**DOI:** 10.1242/bio.20149407

**Published:** 2014-10-10

**Authors:** Sabarish Nagarajan, Savraj S. Grewal

**Affiliations:** Department of Biochemistry and Molecular Biology, and Clark H. Smith Brain Tumour Centre, Southern Alberta Cancer Research Institute, University of Calgary, HRIC, 3330 Hospital Drive NW, Calgary, AB T2N 4N1, Canada

**Keywords:** *Drosophila*, TOR, Growth control, Insulin, mRNA translation, Nutrition

## Abstract

The larval period of the *Drosophila* life cycle is characterized by immense growth. In nutrient rich conditions, larvae increase in mass approximately two hundred-fold in five days. However, upon nutrient deprivation, growth is arrested. The prevailing view is that dietary amino acids drive this larval growth by activating the conserved insulin/PI3 kinase and Target of rapamycin (TOR) pathways and promoting anabolic metabolism. One key anabolic process is protein synthesis. However, few studies have attempted to measure mRNA translation during larval development or examine the signaling requirements for nutrient-dependent regulation. Our work addresses this issue. Using polysome analyses, we observed that starvation rapidly (within thirty minutes) decreased larval mRNA translation, with a maximal decrease at 6–18 hours. By analyzing individual genes, we observed that nutrient-deprivation led to a general reduction in mRNA translation, regardless of any starvation-mediated changes (increase or decrease) in total transcript levels. Although sugars and amino acids are key regulators of translation in animal cells and are the major macronutrients in the larval diet, we found that they alone were not sufficient to maintain mRNA translation in larvae. The insulin/PI3 kinase and TOR pathways are widely proposed as the main link between nutrients and mRNA translation in animal cells. However, we found that genetic activation of PI3K and TOR signaling, or regulation of two effectors – 4EBP and S6K – could not prevent the starvation-mediated translation inhibition. Similarly, we showed that the nutrient stress-activated eIF2α kinases, GCN2 and PERK, were not required for starvation-induced inhibition of translation in larvae. These findings indicate that nutrient control of mRNA translation in larvae is more complex than simply amino acid activation of insulin and TOR signaling.

## INTRODUCTION

Nutrient availability is an important determinant of growth and survival during animal development. When abundant, nutrients promote metabolic processes to drive tissue and body growth. In contrast, under nutrient deprivation, animals need to alter their metabolism to limit energetically costly processes, restrict growth and promote survival.

One important metabolic process controlled by nutrients is mRNA translation. Translation is estimated to account for 30% of ATP use in animal cells (Buttgereit and Brand, 1995). When considered with the energetic cost of synthesizing ribosomes, it is clear that protein synthesis represents the main metabolic activity in growing and proliferating cells. Hence, tight regulation of mRNA translation is essential for cells, tissues and organisms to maintain proper homeostasis. Defects in this regulation often lead to pathologies such as cancer, growth disorders, diabetes and obesity (Sonenberg and Hinnebusch, 2007).

Our knowledge of how nutrients regulate mRNA translation in animal cells comes predominantly from work in tissue culture. These studies have identified two main signaling pathways that link nutrients to the control of mRNA translation:

1. The first is dependent on TORC1, a protein complex containing the conserved serine/threonine kinase target of rapamycin (TOR) (Ma and Blenis, 2009). TORC1 is activated by a signaling network that responds to both extracellular nutrients, such as glucose and amino acids, and to endocrine signaling via insulin/insulin-like growth factors (IGFs), which are controlled by dietary nutrients (Wullschleger et al., 2006). The current view is that TORC1 promotes mRNA translation by phosphorylating and inhibiting 4EBP, a translational repressor that normally binds to the mRNA cap-binding protein eIF4E and prevents it from initiating translation (Pause et al., 1994; Proud, 2007; Sonenberg and Hinnebusch, 2007; Ma and Blenis, 2009; Proud, 2009; Sonenberg and Hinnebusch, 2009). This mechanism is widely proposed to explain how TOR controls cell growth and proliferation in cell culture (Dowling et al., 2010). In addition, TORC1 can phosphorylate another kinase, S6K, which phosphorylates several eukaryotic initiation factors (eIFs) to promote translation (Ma and Blenis, 2009).
2. The second pathway involves phosphorylation of eIF2α (Farrell et al., 1977). When GTP-bound, eIF2α associates with tRNAiMet to form the ternary complex essential for translation initiation. However, phosphorylation keeps eIF2α in an inactive GDP-bound state, thus inhibiting translation. Two kinases mediate the phosphorylation of eIF2α in response to nutrient changes (Spriggs et al., 2010). The first is GCN2, which becomes activated upon amino acid starvation to suppress mRNA translation (Hinnebusch, 1994; Proud, 2014). The second is PERK, which is activated by ER stress – a response that can occur as a result of abnormal glucose levels (Kaufman et al., 2002; Ron and Harding, 2012).

Many hundreds of studies have examined how these two signaling pathways regulate mRNA translation in tissue culture. In particular, this work has emphasized the importance of both pathways in linking changes in nutrients and insulin/insulin-like growth factors to the translational regulation of growth and metabolic homeostasis in cells (Sonenberg and Hinnebusch, 2007). However less is known about how both pathways contribute to nutrition-regulated protein synthesis in animals.

*Drosophila* larvae have provided a versatile model for examining how nutrients control metabolism and growth during animal development. Following embryogenesis, larvae hatch and begin feeding. Over the next 4–5 days they increase in mass almost two hundred fold (Church and Robertson, 1966), before pupae formation and metamorphosis into adults. This massive increase in larval growth is dependent on both endocrine insulin signaling and activation of the TORC1 signaling pathway (Oldham and Hafen, 2003; Grewal, 2009; Teleman, 2010). In rich nutrient conditions, both pathways are activated, leading to cell, tissue and body growth. In contrast inhibition of insulin or TORC1 signaling, either by nutrient deprivation or genetic mutation leads to organismal growth arrest. Compared to insulin and TORC1 less is known about the role for GCN2 in larval development, although one report has demonstrated a role for the kinase in mediating changes in feeding behaviour in response to amino acid imbalanced diets (Bjordal et al., 2014).

Regulation of protein synthesis has been shown to be important for controlling larval growth. Mutants for ribosomal proteins or various translation factors exhibit growth defects (Galloni and Edgar, 1999; Lachance et al., 2002; Marygold et al., 2007), and feeding larvae with chemical inhibitors of translation leads to growth arrest (Britton and Edgar, 1998). Several reports have also described how nutrient availability and both insulin and TORC1 signaling can regulate synthesis and activation of various components of the translation machinery such as rRNA, tRNA, and ribosome biogenesis and translation initiation factors (Miron et al., 2001; Lachance et al., 2002; Arquier et al., 2005; Grewal et al., 2005; Reiling et al., 2005; Grewal et al., 2007; Teleman et al., 2008; Li et al., 2010; Marshall et al., 2012). However, few reports have attempted to measure mRNA translation in *Drosophila* larvae and examine both the specific nutrient and signaling requirements for maintaining translation. We address this issue in this paper.

## MATERIALS AND METHODS

### Egg collection

Eggs were collected from adult flies for a period of 4–6 hours on grape juice agar plates supplemented with yeast paste. The next day hatched larvae were transferred to vials (50 larvae per vial).

### Drosophila stocks

w^1118^, yw, ywhsflp^122^, tor^Δ6B^, UAS-Dp110^WT^, UAS-Rheb, act>CD2>GAL4, UAS-GFP, thor^2^, UAS-S6K^TE^, daGAL4, UAS-GCN2-IR (Bloomington Stock Center, TRiP collection, #35355), UAS-GCN2-IR (NIG stock Center, Kyoto, #1609), UAS-PEK IR (Bloomington Stock Center, TRiP collection, #35162). For all GAL4/UAS experiments, homozygous GAL4 lines were crossed to the relevant UAS line(s) and the larval progeny were analyzed. Control animals were obtained by crossing the relevant homozygous GAL4 line to either w^1118^; +; + or yw; +; +, depending on the genetic background of the particular experimental UAS transgene line.

### Food conditions

Larvae were grown on our standard laboratory food: 150 g agar, 1500 g cornmeal, 770 g yeast, 675 g sucrose, 1875 g D-glucose, 240 ml propionic acid per 34.5 L water.

**Fig. 1.**
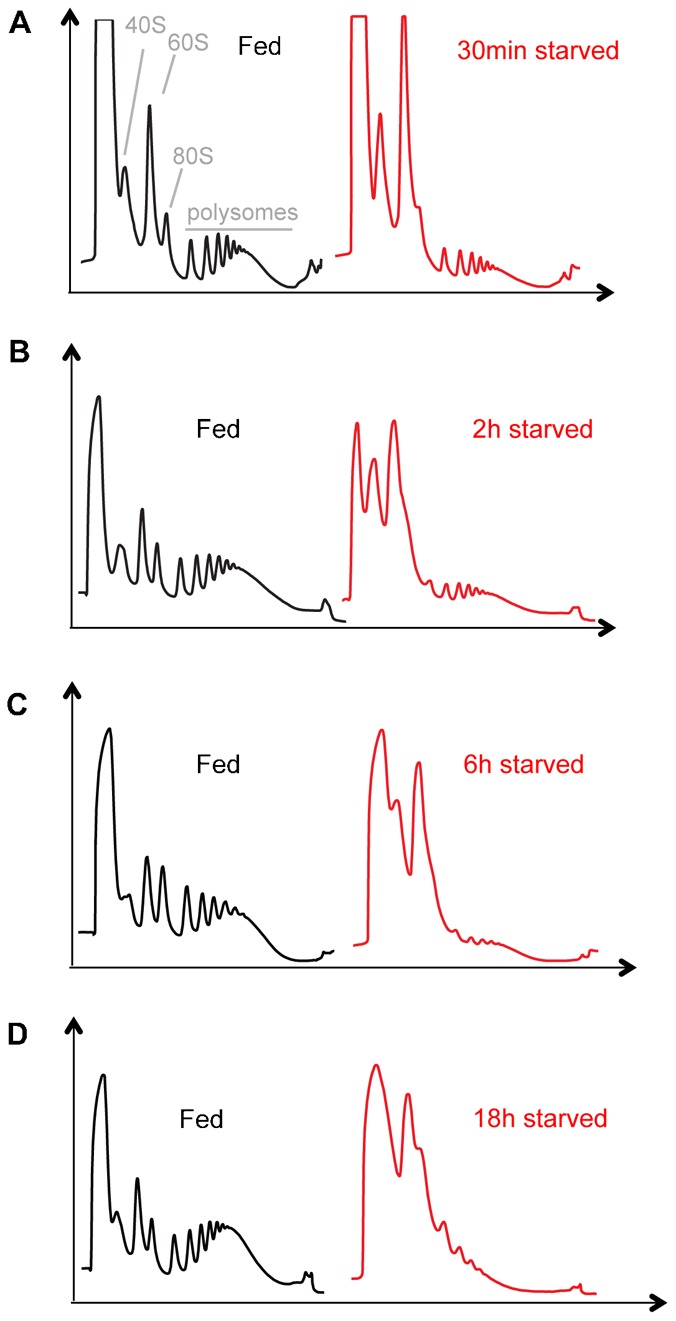
Starvation leads to a rapid inhibition of mRNA translation in *Drosophila* larvae. Representative polysome traces for experiments in which we examined the effects of starvation (floating larvae on a sugar: PBS solution) for (A) 30 minutes, (B) 2 hours, (C) 6 hours, and (D) 18 hours. In each experiment the starvation profiles were compared to larvae maintained on normal laboratory food. The relative positions of the 40S, 60S, 80S and polysomes are indicated in panel A.

**Fig. 2.**
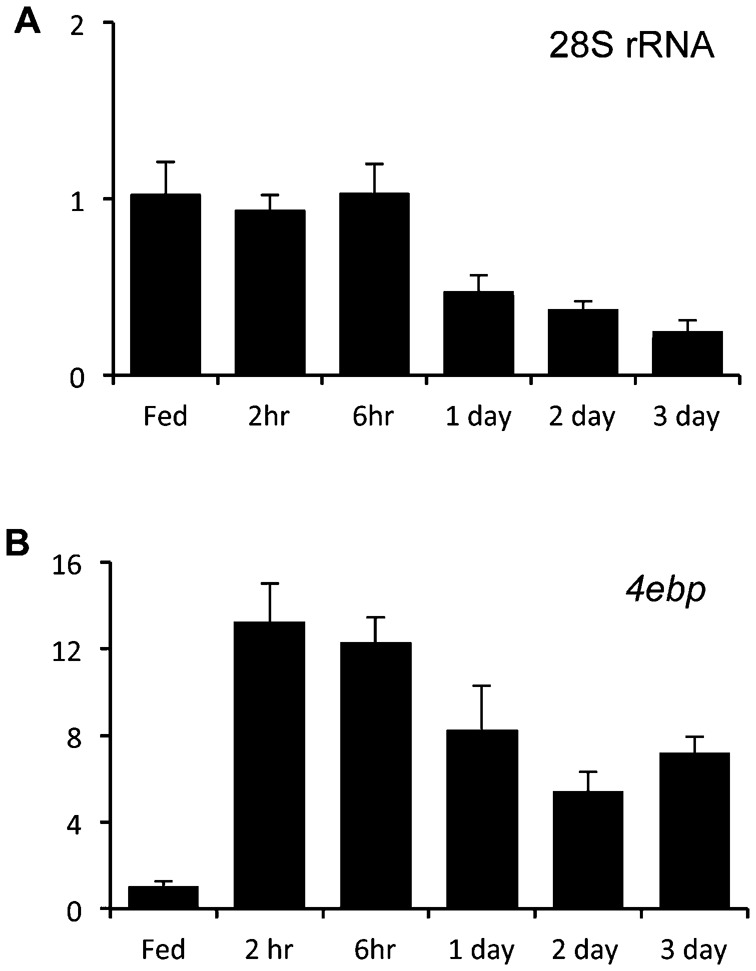
Starvation leads to reduced ribosome numbers in larvae. Data from qPCR analysis of (A) 28S rRNA, and (B) 4ebp mRNA levels in 72 hr old fed and starved wild-type larvae. Starvation was achieved by floating larvae on a sugar: PBS solution for starvation periods of 2 hrs to 3 days. Data represent mean ± SEM values (N = 5 per timepoint).

**Fig. 3.**
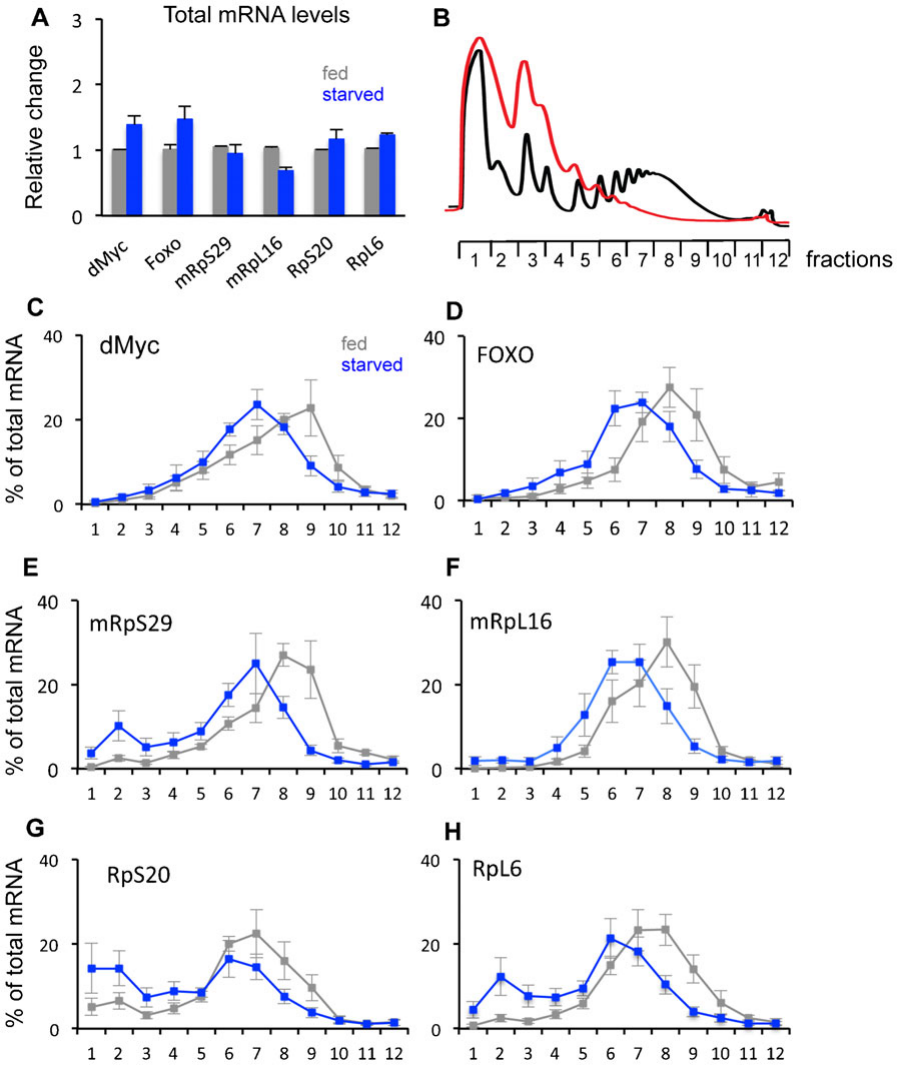
Translational control of genes whose total mRNA levels are unchanged by starvation. (A) Total mRNA levels of each of the six genes were measured by qRT-PCR in fed vs. 18 hr starved larvae. Data are presented as mean ± SEM. (B) Schematic showing the twelve polysome fractions used for qRT-PCR analysis. The traces indicate representative polysome profiles from fed (black trace) and 18 hr starved (red trace) larvae. The twelve fractions processed for RNA extraction and qRT-PCR analysis are indicated. (C–H) qRT-PCR analysis of each of the six selected genes. Each data point in the figure shows the mean (± SEM) % of total mRNA in each of the twelve fractions. Grey bars, fed larvae; blue bars, starved larvae.

**Fig. 4.**
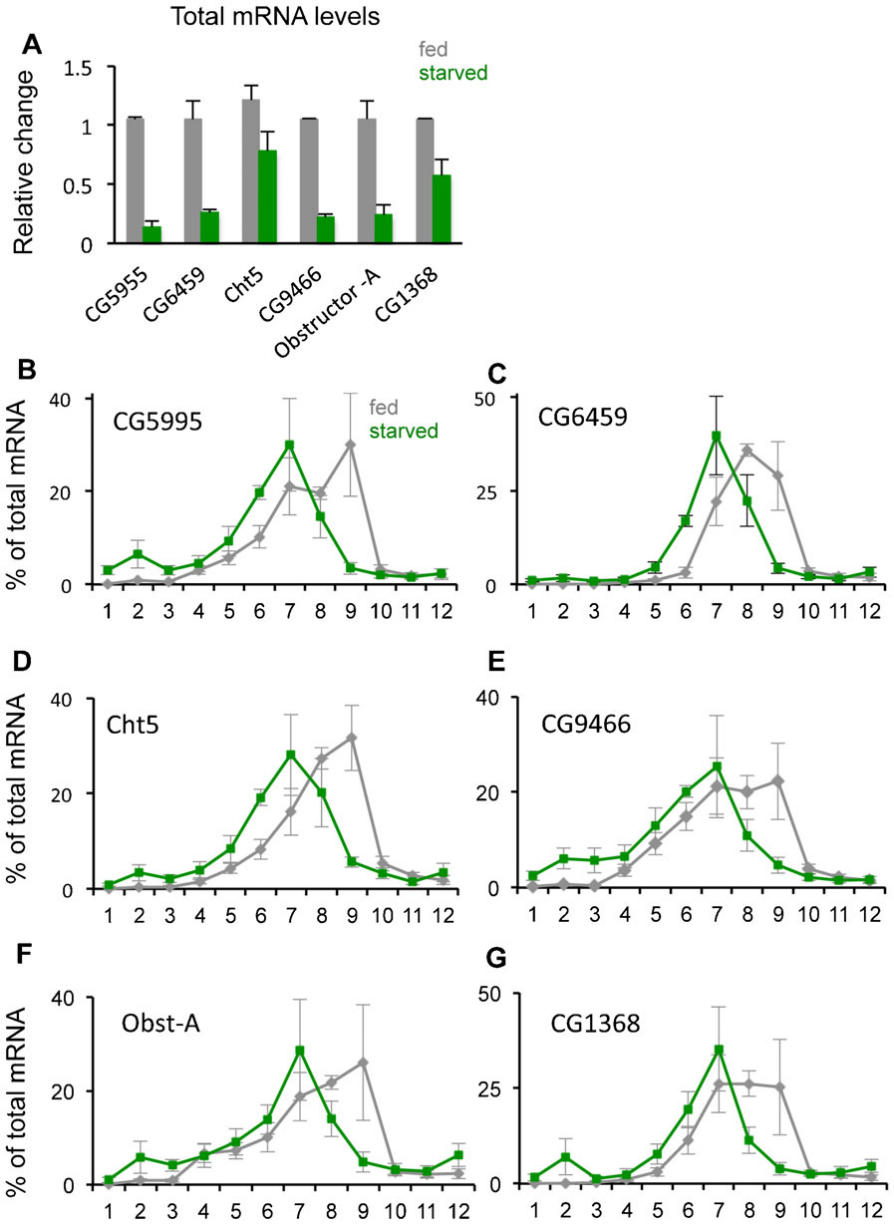
Translational control of genes whose total mRNA levels are decreased by starvation. (A) Total mRNA levels of each of the six genes were measured by qRT-PCR in fed vs. 18 hr starved larvae. Data are presented as mean ± SEM. (B–G) qRT-PCR analysis of each of the six selected genes. Each data point in the figure shows the mean (± SEM) % of total mRNA in each of the twelve fractions. Grey bars, fed larvae; green bars, starved larvae.

**Fig. 5.**
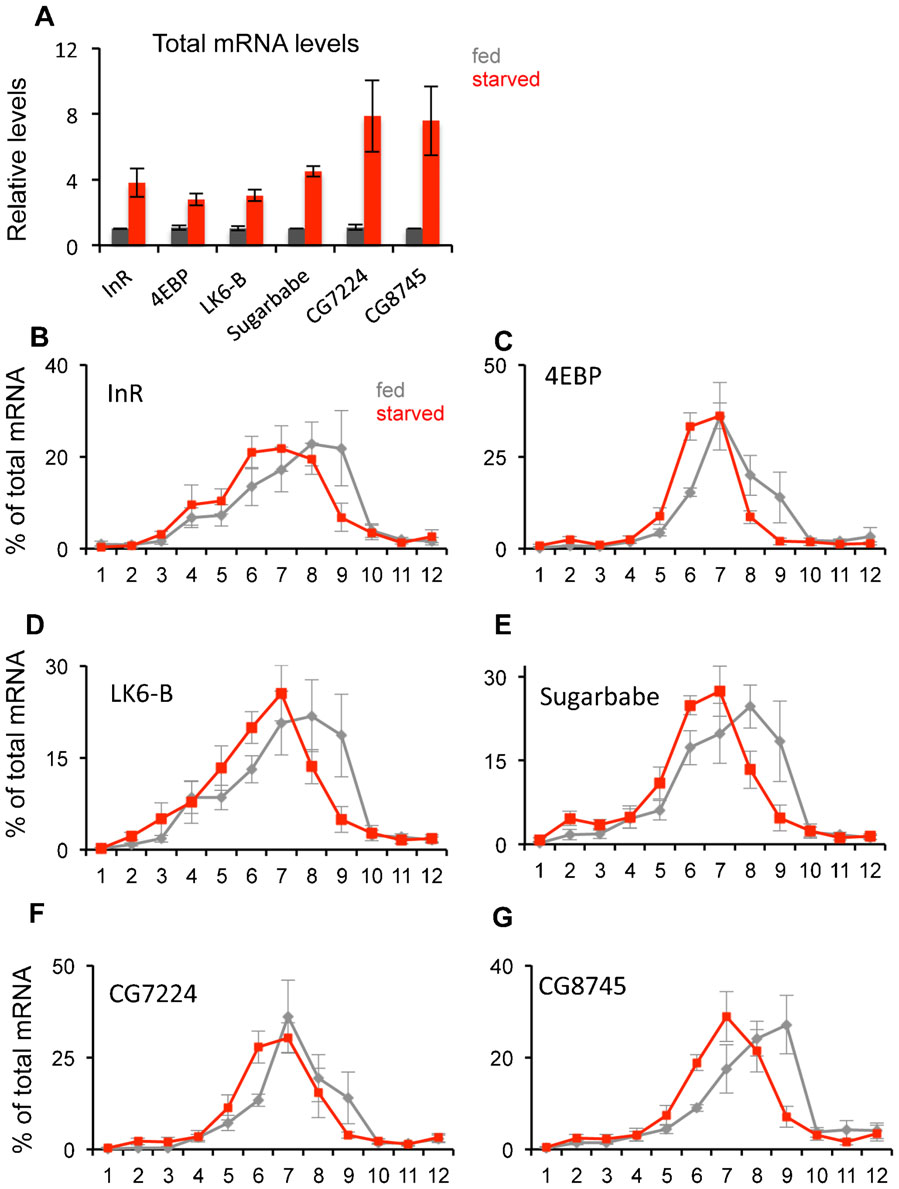
Translational control of genes whose total mRNA levels are increased by starvation. (A) Total mRNA levels of each of the six genes were measured by qRT-PCR in fed vs. 18 hr starved larvae. Data are presented as mean ± SEM. (B–G) qRT-PCR analysis of each of the six selected genes. Each data point in the figure shows the mean (± SEM) % of total mRNA in each of the twelve fractions. Grey bars, fed larvae; red bars, starved larvae.

**Fig. 6.**
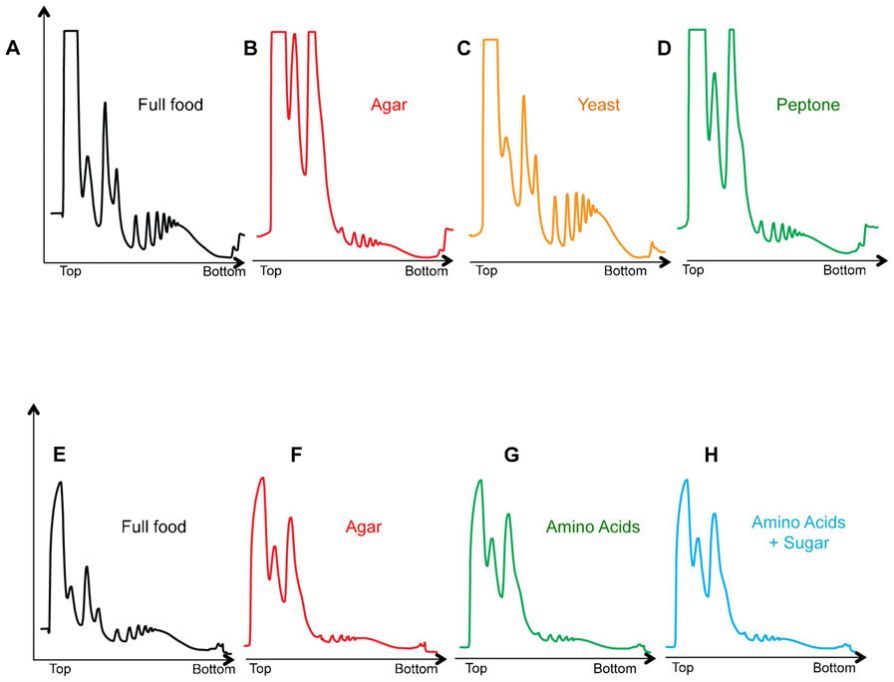
Yeast, but not amino acids or sugar, are sufficient for mRNA translation in larvae. Representative polysome traces for experiments in which we examined the effects of different food conditions on mRNA translation. In each case, 72 hr old larvae were transferred from our normal laboratory food to one of the indicated diets. Experiment one (A–D): larvae were transferred to (A) full food, (B) agar alone, (C) 30% yeast: agar starvation, or (D) 15% peptone: agar. Experiment two (E–H): larvae were transferred to (E) full food, (F) agar alone, (G) an amino acid: agar food containing all twenty amino acids (see Materials and Methods for concentrations), or (H) amino acid: sugar: agar food (see Materials and Methods for concentrations).

**Fig. 7.**
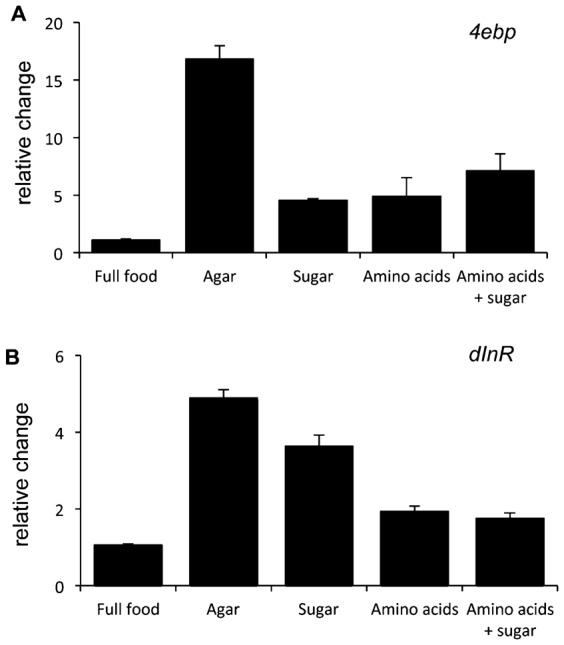
Amino acids plus sugar can strongly reverse the starvation-mediated increase in 4ebp and dInR mRNA levels. Data from qPCR analysis of (A) 4ebp mRNA, and (B) dInR mRNA levels in 72 hr old wild-type larvae 2 hrs after transferring them from lab food to full food, agar alone, agar:sugar, agar:amino acids, or agar:amino acids:sugar (see Materials and Methods for nutrient concentrations). Data represent mean ± SEM values (N = 5 per condition).

**Fig. 8.**
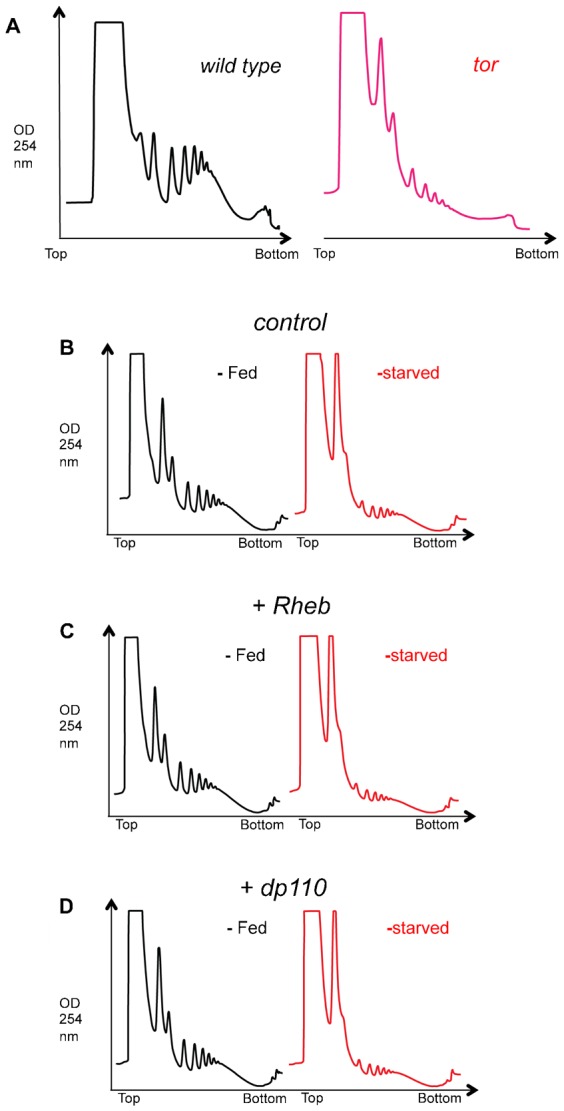
TORC1 and insulin regulation of mRNA translation. (A) Representative trace from an experiment comparing polysome profiles in wild-type (left trace, black) versus *tor* mutant (right trace, red) larvae. Genotypes: wildtype, *w^1118;;^*; *tor* mutant, *w^1118^;tor^ΔP^/tor^ΔP^*. (B–D) Polysome profiles from (B) control, (C) Rheb-overexpressing, (D) dp110-overexpressing larvae. Black traces from fed larvae, red traces from starved larvae. Genotypes – control: *ywhsflp^122^/+; +/+; act>CD2>Gal4, UAS-GFP/+*, Rheb overexpression: *ywhsflp^122^/+; UAS-Rheb/+; act>CD2>Gal4, UAS-GFP/+*, dp110 overexpression: *ywhsflp^122^/+; UAS-dp110/+; act>CD2>Gal4, UAS-GFP/+*. Transgene expression was induced at 48 hr after egg laying. Larvae were then starved (sucrose: PBS) at 72 hr after egg laying.

**Fig. 9.**
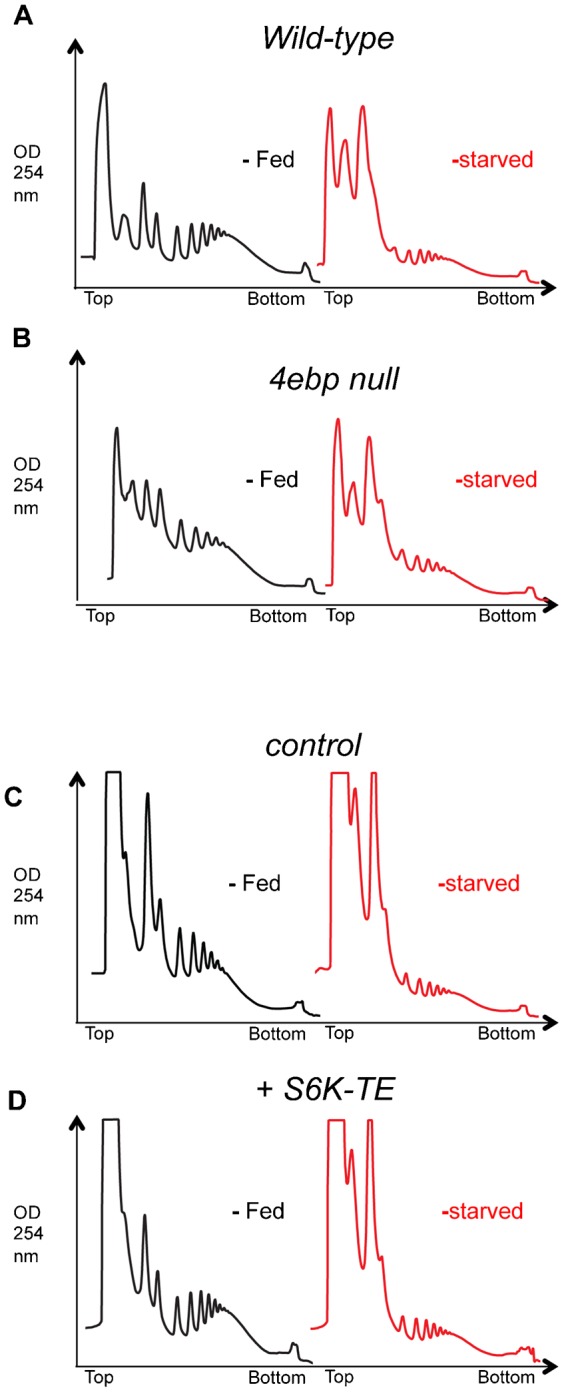
4EBP and S6K do not regulate bulk translation in larvae. (A,B) Polysome profiles in (A) wild-type versus (B) *thor* mutant larvae. Black traces from fed larvae, red traces from starved (sucrose: PBS) larvae. Genotypes: wildtype, *yw^1118;;^*; *4ebp* mutant, *yw^1118^;thor^1^/thor^1^*. (C,D) Polysome profiles from (C) control, and (D) S6K-TE-overexpressing larvae. Black traces from fed larvae, red traces from starved (sucrose: PBS) larvae. Genotypes - control: *da-GAL4/+*, S6K-TE overexpression: *UAS-S6K-TE/+; da-GAL4/+*.

For starvation experiments (Figs 1–5 and 7–9) larvae were removed from from fly food at 72 hours after egg-laying, washed and transferred to a 20% sucrose: PBS solution. For prolonged starvation, fresh 20% sucrose was replaced each day. For the experiments shown in Fig. 6, larvae were removed from food at 72 hours after egg laying and transferred to one of the following foods:

Yeast only: 15% yeast in 1× PBS (pH 7.4), 0.5% Agar.

Agar only: 1× PBS, 0.5% Agar.

Peptone only: 15% Peptone in 1× PBS, 0.5% Agar.

Total amino acids: L-Phenylalanine – 0.7 g/l, L-Histidine – 0.6 g/l, L-Lysine – 1.5 g/l, L-Methionine – 0.3 g/l, L-Arginine – 1.2 g/l, L-Threonine – 1.1 g/l, L-Valine – 1.1 g/l, L-Tryptophan – 0.4 g/l, L-Isoleucine – 1 g/l, L-Leucine – 1.4 g/l, L-Glycine – 0.6 g/l, L-Alanine – 0.8 g/l, L-Asparagine – 1.1 g/l, L-Aspartic Acid – 1.1 g/l, L-Glutamic Acid – 1.2 g/l, L-Proline – 1 g/l, L-Serine – 0.9 g/l, L-Cysteine – 0.2 g/l, L-Tyrosine – 0.7 g/l, L-Glutamine – 1.5 g/l in 1× PBS, 0.5% Agar.

Total amino acids + sugar: Amino acids as in Total amino acids alone + 2.11% sucrose, 7.3125% D-glucose in 1× PBS, 0.5% Agar.

### Polysome gradient centrifugation

200 larvae (4 vials with 50 larvae/vial) were quickly washed with filter sterilized 20% sucrose PBS and lysed in 700 µl of lysis buffer (25 mM Tris pH 7.4, 10 mM MgCl_2_, 250 mM NaCl, 1% Triton X-100, 0.5% sodium deoxycholate, 0.5 mM DTT, 100 mg/ml cycloheximide, 1 mg/ml heparin, 1× Complete mini roche protease inhibitor, 2.5 mM PMSF, 5 mM sodium fluoride, 1 mM sodium orthovanadate and 200 U/ml ribolock RNAse inhibitor (Fermentas) using a Dounce homogenizer. The lysates were centrifuged at 15,000 rpm for 20 minutes and the supernatant was removed carefully using a fine syringe to avoid the floating fat content. 300 µg RNA was layered gently on top of a 15–45% w/w sucrose gradient (made using 25 mM Tris pH 7.4, 10 mM MgCl_2_, 250 mM NaCl, 1 mg/ml heparin, 100 mg/ml cycloheximide in 12 ml polyallomer tube) and centrifuged at 37,000 rpm for 150 minutes in a Beckmann Coulter Optima L-90K ultracentrifuge using a SW-41 rotor. Polysome profiles were obtained by pushing the gradient using 70% w/v Sucrose pumped at 1.5 ml/min into a continuous OD254 nm reader (ISCO UA6 UV detector) showing the OD corresponding to the RNA present from the top to the bottom of the gradient.

### Polysomal and total mRNA analyses

For the quantification of transcript levels in the polysome gradients, the 12 ml sucrose gradient was divided into 12 fractions of 1 ml each. The protein was denatured by addition of 1/100 volume of 10% SDS and the RNA was then precipitated by adding 1/50 volume of 5M NaCl and 2.5 volumes of ethanol at −20°C for 2 hours or kept overnight. The RNA was then centrifuged at 16,000G for 20 minutes at 4 degrees. The supernatant was discarded and the pellet was resuspended in 150 µl RNAse free water. The RNA was then extracted as per the manufacturer's protocol (TRIzol) and then resuspended in 100 µl DNAse and RNAse free water. To remove any traces of Heparin, the resuspended RNA was precipitated by adding 1 volume of 3M Lithium chloride, kept overnight at −20°C and centrifuged at 13,000 rpm for 15 minutes. The supernatant was discarded and the pellets were washed with 75% ethanol to remove any LiCl. The pellets were resuspended in 100 µl DNAse and RNAse free water. The resuspended RNA was treated with DNAse (Ambion).

For analysis of total RNA levels, RNA was extracted from whole larvae using TRIzol according to the manufacturer's instructions. For both total RNA and polysomal RNA, cDNA was made using the Superscript III reverse transcriptase (Life Technologies) as per the manufacturer's instructions. The cDNA was used as a template to perform qRT-PCR reactions (BioRad Laboratories, MyIQ PCR machine using SyBr Green PCR mix) using specific primer pairs (sequences available upon request). For experiments in which we anlayzed total mRNA levels, data was normalized to β*Tubulin* mRNA levels.

## RESULTS

We used polysome profiling to examine how mRNA translation was influenced by nutrient availability in *Drosophila* larvae. In this assay whole larval lysates were subjected to sucrose density centrifugation. This allowed separation and visualization of ribosomes engaged in translation (polysomal fraction) or individual monosomes (80 s) and subunits (40S, 60S). In each experiment we centrifuged lysates with equivalent RNA levels. Since most RNA is ribosomal, this process ensured that we could directly compare between conditions to estimate the proportion of ribosomes engaged in translation (polysomes), independent of any experimentally induced change in total ribosome numbers.

### Starvation induces a rapid inhibition of mRNA translation

Larval growth is dependent on dietary nutrients, especially protein and amino acids. Upon hatching, larvae require dietary protein to initiate cell cycle progression and cell growth in all larval tissues (Britton and Edgar, 1998). Starvation for dietary protein in young larvae leads to an organismal growth arrest. This starvation and growth arrest can be achieved by removing larvae from food and floating them on a sucrose/PBS solution (Britton and Edgar, 1998; Britton et al., 2002). We used this protocol to examine the effects of starvation on polysomes in 72 hr larvae – the approximate midpoint of the larval growth period.

We found that starvation led to a reduction in the proportion of ribosomes engaged in translation and a concomitant increase in free 40S, 60S and 80S ribosome subunit levels (Fig. 1). These effects were consistent with a reduction in translation initiation. This decrease in translation was apparent as early as 30 minutes following starvation (Fig. 1A), and was pronounced by 2 hr of starvation (Fig. 1B). Reduction in polysome levels reached a maximal low between 6 and 18 hrs (Fig. 1C,D). Starvation for longer periods (we measured up to 4 days) showed this similar low level of translation (data not shown).

We also used qRT-PCR to measure total rRNA levels (as an index of ribosome numbers) upon starvation. We found that at the earlier starvation time points (2–6 hrs) levels of 28S rRNA were unchanged compared to fed larvae (Fig. 2A). Only at starvation time points of 24 hrs and longer were rRNA levels reduced. In contrast, we observed that levels of 4EBP mRNA were higher at all starvation time points (Fig. 2B). 4EBP is induced by the transcription factor FOXO when insulin signaling is suppressed by starvation (Jünger et al., 2003). Hence, this result confirms that all time points exhibited a strong starvation-mediated gene expression response. These data suggest that upon starvation, translation initiation is inhibited as early as 30 minutes and that ribosome numbers become limiting only after longer periods of starvation.

To examine these starvation effects further, we analyzed translation of specific mRNAs by measuring their association with polysomes. We previously used microarray analyses to explore genome-wide transcript changes in response to starvation in larvae (Li et al., 2010). We used this data set to select 18 mRNAs to test for translational changes – six of these mRNAs showed little or no change in total levels upon starvation, six showed a marked increase, and six showed a decrease. The analyses for the three sets of genes are shown in Figs 3–5. We first confirmed the starvation-mediated changes in total mRNA levels using qRT-PCR analysis, and in general found good agreement with our microarray analyses (Fig. 3A, Fig. 4A, Fig. 5A). Although by no means an exhaustive set of mRNAs, the selection of these 18 genes allowed us to a) examine how translation of individual mRNAs responds to starvation, and b) identify any potential correlation between changes in total transcript levels versus specific changes in translation. To perform the translation analyses, we selected an 18 hr starvation time point and following sucrose density centrifugation, we divided the gradient contents into 12 equal fractions and performed qRT-PCR to measure mRNA levels in each fraction (Fig. 3B). Two general themes emerged from this analysis. First, in fed animals, for 16 out of 18 genes, most mRNA was found in fractions 7–9. This corresponds to a polysome containing 5–8 ribosomes. The remaining two genes (4EBP and CG7224 – Fig. 5C,F) are both small genes, which may limit the numbers of ribosomes that can associate with their mRNAs. These polysome data suggest that translation is generally at a high level in feeding larvae. Second, we observed that upon starvation, for almost all genes the peak of mRNAs shifted to fractions 5–7, which contains polysomes with 2–5 ribosomes. These effects were seen regardless of whether total mRNA levels for the genes were unchanged (Fig. 3), downregulated (Fig. 4) or upregulated (Fig. 5). Hence, even upon starvation almost all mRNAs are still polysomal, albeit with a shift in polysome association consistent with reduced translation. With the exception of two RP mRNAs, we saw little or no increase in mRNAs in fractions 1–5 upon starvation. These fractions contain untranslated mRNAs (e.g. mRNAs associated with mRNP complexes or sequestered in P-bodies). Together these findings suggest that translation of all mRNAs was reduced, but not abolished, upon starvation, regardless of the change in total mRNA levels.

### Neither dietary amino acids nor sugars are sufficient to maintain mRNA translation

We next explored the nutrient requirements for maintaining mRNA translation in larvae. *Drosophila* larvae are typically raised on a laboratory diet with sugars and yeast as the main nutrient sources. In each of the following experiments, we transferred larvae from this food into different conditions for two hours to identify the nutrient requirements to maintain mRNA translation. In all our experiments, we removed larvae from their food by manually dissociating and floating them out of the food using a 20% sucrose solution, before washing and transferring to a new food. When we did this and then simply returned the larvae back to food, we observed that the polysome profile was identical to a normal fed larvae profile (Fig. 6A,E). This result indicates the mechanical process of transferring larvae between food sources had no effect on mRNA translation. When we transferred larvae from food to agar alone we observed a reduction in mRNA translation, consistent with the starvation effects described above (Fig. 6B,F). Although diets vary considerably from laboratory to laboratory, in almost all cases, yeast provides the major nutrient source. When larvae were transferred to an agar: yeast food source, we found that translation was maintained at a level comparable to fully fed larvae maintained on our laboratory food (Fig. 6C).

Two main nutrient sources that may be important for translation are sugars and amino acids. We therefore tested whether either or both were important for translation in larvae. Our initial findings above showed that transferring larvae to a sucrose: PBS food led to a reduction in mRNA translation similar to that seen with a complete (agar alone) food. Thus, sugars alone are not enough to maintain translation. We therefore tested amino acids. We first performed an experiment in which we transferred larvae to an agar + peptone food source, which provided a rich source of dietary protein, similar or greater than that present in yeast. We found that larvae transferred to this peptone diet showed a reduction in polysome levels similar to that seen in complete starvation (Fig. 6D). Similar effects were seen when we used casamino acids as the protein source (data not shown). We further examined an agar + complete amino acid diet, in which we provided defined amounts of all twenty amino acids based on a holidic diet previously shown to support *Drosophila* development (Piper et al., 2014). Again, we observed that providing amino acids alone was not sufficient to maintain translation to a level seen in fed animals (Fig. 6G). A combined food of peptone + all essential amino acids also had no effect on the starvation induced decrease in mRNA translation (data not shown). Finally, we examined a combined agar + peptone + sugar or agar + amino acid + sugar diet, and found that in both cases polysome levels were suppressed to the same degree as complete starvation (Fig. 6H). These data suggest that neither amino acids nor sugars alone or together are sufficient to maintain mRNA translation in larvae. However, we did see that amino acid and sugars were sufficient to blunt the starvation mediated increase in both *4ebp* and *dInR* mRNA, two transcripts that are induced by FOXO when insulin signaling is suppressed by starvation (Fig. 7).

### Insulin/TOR signaling is necessary but not sufficient to maintain nutrient-dependent mRNA translation

We next explored the signaling requirements for maintaining nutrient-dependent mRNA translation in larvae. The insulin and TORC1 kinase signaling pathways are the major nutrient-dependent regulators of cell, tissue and body growth in *Drosophila* larvae. Furthermore, an extensive body of work using tissue culture shows that TORC1 signaling is a link between nutrients and mRNA translation in animal cells. We therefore examined if insulin/TORC1 signaling regulates mRNA translation in larvae. We first examined mRNA translation in *tor* null mutants. Using the same polysomal profiling technique described above, we found that compared to wild type larvae, *tor* null larvae had markedly reduced levels of polysomes and an increase in subpolysomal fractions (Fig. 8A). These effects phenocopied the changes in translation seen in starved larvae.

Nutrient availability activates insulin and TORC1 signaling in larvae and promotes growth. Thus, upon starvation, one possibility is that a loss of insulin/TORC1 signaling leads to suppression of mRNA translation. Therefore, a prediction is that maintaining insulin/TORC1 signaling in starved animals may be sufficient to keep translation high. To test this possibility, we examined the effects of genetically activating both the TORC1 and insulin pathways. We first examined overexpression of Rheb, the upstream activator of TORC1. We used the *hsflp-out/actin-GAL4* system to express a *UAS-Rheb* transgene ubiquitously in second instar larvae (which led to a 3.4±0.6 fold induction of rheb mRNA as measured by qRT-PCR) and examined effects on polysomes. We previously showed that using this approach, overexpression of Rheb could increase protein synthesis in larvae, as measured by an *ex vivo* tritiated amino acid incorporation assay (Hall et al., 2007). Furthermore, we showed that one potential mechanism for this effect was stimulation of ribosome synthesis (Grewal et al., 2007). Here we examined the effects of Rheb overexpression on polysome levels in both fed and starved larvae. We found that Rheb overexpression had no marked effect on the ratio of polysomes to subpolysomes in fed larvae compared to control animals (Fig. 8B,C). Given our previous result showing increased overall protein synthesis in Rheb-overexpressing larvae, this result suggests that Rheb works by either simultaneously promoting both initiation and elongation (thus maintaining the ratio of polysomes to sub polysomes) or that the predominant mechanism of Rheb/TORC1-induced protein synthesis in larvae involves an increase in overall ribosome numbers without necessarily any change in the proportion of ribosomes engaged in translation.

We next examined the effects of Rheb-overexpression in starved larvae. Again we used the flp-out system to express *UAS-Rheb* and then transferred larvae to a sugar only diet. In contrast to the prediction above, Rheb expression was not sufficient to maintain mRNA translation in larvae starved for either 2 hr (data not shown) or 6 hr (Fig. 8B,C). We also examined the effects of increasing TORC1 by using RNAi to knockdown levels of TSC1, a negative regulator of TORC1 signaling (Gao and Pan, 2001; Potter et al., 2001; Tapon et al., 2001). We expressed a *UAS-tsc1 IR* (inverted repeat) construct with the ubiquitous *da-GAL4* driver. As with Rheb overexpression, we found that knockdown of TSC1 did not alter the polysome: subpolysome ratio in fed larvae, and did not maintain translation under starvation (data nor shown). Finally, we examined the effects of maintaining high levels of insulin signaling. To achieve this, we used the *hsflp-out/actin-GAL4* system to overexpress *UAS-dp110* (30±3 fold induction of *dp110* mRNA as measured by qRT-PCR), the catalytic subunit of PI3 kinase, which functions downstream of the insulin receptor. We observed similar effects to those with seen with Rheb overexpression: dp110 overexpression did not affect the polysome: subpolysome ratio in fed larvae and did not prevent the starvation-mediated repression of mRNA translation (Fig. 8D).

The translational repressor 4EBP and the kinase S6K are two effectors of TORC1 that have been extensively studied in tissue culture as regulators of mRNA translation. 4EBP is phosphorylated and inhibited by TORC1 to allow translation initiation (Ma and Blenis, 2009). S6K is also phosphorylated by TORC1, and in turn it phosphorylates and augments the activity of several translation factors (Ma and Blenis, 2009). We therefore examined a role for these two effectors in mRNA translational control in larvae. We first found that in fed conditions *4ebp* null larvae showed no increase in polysome levels compared to wild type larvae (Fig. 9A,B). Furthermore, the starvation-mediated repression of mRNA translation seen in wild type larvae was still maintained in *4ebp* null animals (Fig. 9A,B). We next examined the effect of S6K on mRNA translation. For these experiments we tested the effects of ubiquitous overexpression of a constitutively actively version of S6K (*UAS-S6K-TE*) using the *da-GAL4* driver (6.5±3 fold induction of *s6k* mRNA as measured by qRT-PCR). We saw that *da>S6K-TE* larvae showed no change in polysome levels compared to control (*da > +*) larvae in either fed or starvation conditions (Fig. 9C,D).

Together, these data suggest that although insulin and TORC1 signaling are key mediators of larval growth, maintaining signaling through these pathways or their effectors is not enough to account for nutrient-dependent stimulation of mRNA translation in larvae. These findings are consistent with our earlier findings (Figs 6 and 7) where we saw that amino acids and sugars were sufficient to maintain insulin/PI3K signaling (as indicated by weak induction of *4ebp* and *dInR* mRNA when larvae were transferred to amino acids/sugar compared to the strong induction seen with complete starvation) yet they failed to maintain mRNA translation.

### Activation of GCN2 or PERK does not account for starvation-mediated mRNA translation inhibition

**Fig. 10.**
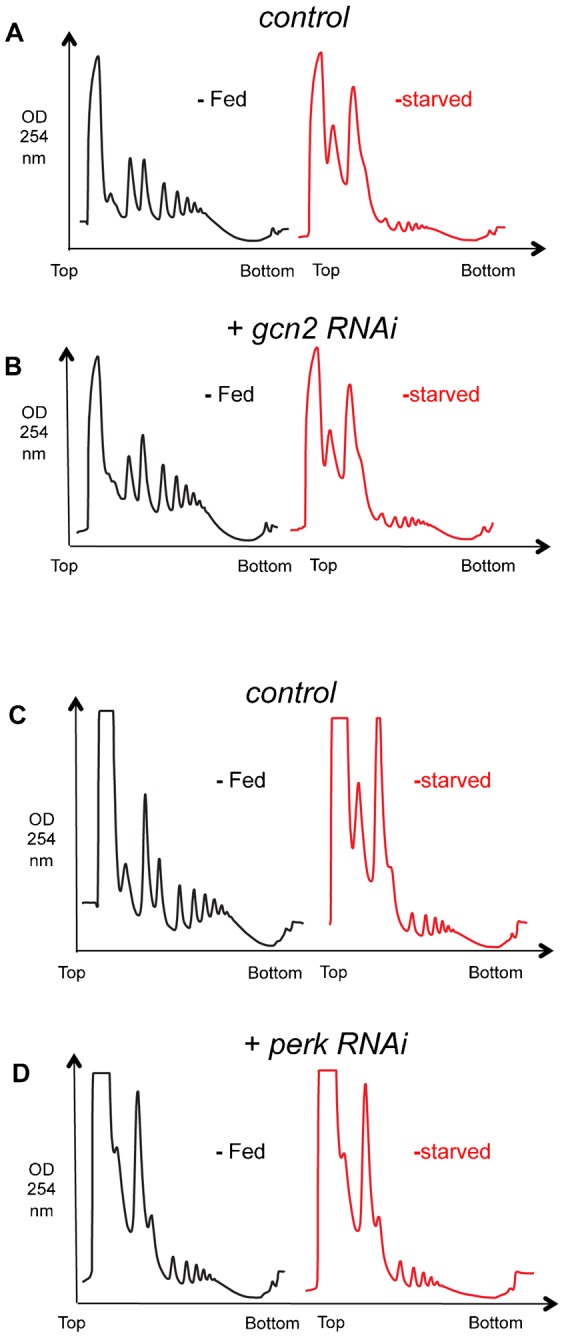
GCN2 and PERK are not involved in the starvation-mediated inhibition of mRNA translation. (A,B) Polysome profiles from (A) control, and (B) GCN2 IR-overexpressing larvae. Black traces from fed larvae, red traces from starved (sucrose: PBS) larvae. Genotypes – control: *da-GAL4/+*, gcn2 RNAi overexpression: *UAS-gcn2 IR/+; da-GAL4/+*. (C,D) Polysome profiles from (C) control, and (D) PERK IR-overexpressing larvae. Black traces from fed larvae, red traces from starved (sucrose: PBS) larvae. Genotypes – control: *da-GAL4/+*, perk RNAi overexpression: *UAS-perk IR/+; da-GAL4/+*.

**Fig. 11.**
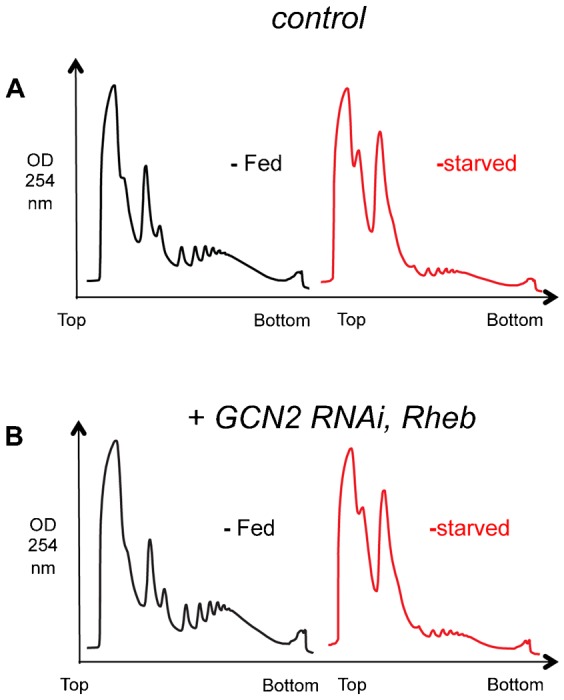
Simultaneous overactivation of TORC1 and knockdown of GCN2 has no effect on starvation-mediated inhibition of bulk mRNA translation. Polysome profiles from (A) control, and (B) Rheb and GCN2 IR-overexpressing larvae. Black traces from fed larvae, red traces from starved larvae. Genotypes – control: *ywhsflp^122^/+; +/+; act>CD2>Gal4, UAS-GFP/+*; Rheb/GCN2 IR overexpression: *ywhsflp^122^/+; UAS-Rheb/UAS-GCN2 IR; act>CD2>Gal4, UAS-GFP/+*. Transgene expression was induced at 48 hr after egg laying. Larvae were then starved (sucrose: PBS) at 72 hr after egg laying.

Amino acid starvation leads to activation of the GCN2 kinase in yeast and animal cells. Active GCN2 then phosphorylates eIF2α, leading to inhibition of mRNA translation initiation. This stress response is thought to explain, in part, how amino acid starvation can lead to translation repression in eukaryotic cells (Hinnebusch, 1994; Sonenberg and Hinnebusch, 2009). We therefore examined a role for *Drosophila* GCN2 in mediating the starvation-induced repression of mRNA translation in larvae. We used RNAi to knockdown GCN2 levels ubiquitously in larvae with a *UAS-GCN2* inverted repeat (Bloomington Stock Center, TRiP collection) driven by *da-GAL4* (*da > GCN2 IR*). This approach lead to a reduction of *gcn2* mRNA to 27.7±2.4% of control as measured by qRT-PCR. We found that under fed conditions *da > GCN2 IR* larvae had slightly higher levels of polysomes compared to control (*da > +*) larvae (Fig. 10A,B). These data suggest that inhibition of GCN2 can promote a modest increase in translation initiation. In contrast, when we starved larvae for 2 hrs, we observed that the repression in mRNA translation in control (*da > +*) larvae was also seen with *da > GCN2 IR* larvae (Fig. 10A,B). Similar results upon starvation were obtained when we used a second, independent UAS-GCN2 RNAi line (data not shown, knockdown of *gcn2* mRNA to 27.6±1.6% of control as measured by qRT-PCR). Furthermore, when we coexpressed UAS-GCN2 IR with UAS-Rheb, to simultaneously maintain TORC1 and inhibit GCN2, we saw no effect on the starvation-mediated inhibition of mRNA translation (Fig. 11). These results suggest that activation of GCN2 does not account for the starvation-mediated inhibition of mRNA translation in larvae.

PERK kinase is another kinase can phosphorylate eIF2α and inhibit mRNA translation (Ron and Harding, 2012). PERK is activated in response to cues that trigger ER stress, including glucose imbalance. We therefore tested a role for PERK activation in mediating starvation-induced mRNA translation. Using a similar approach to our analysis of GCN2, we used RNAi to knockdown PEK, the *Drosophila* homolog of PERK, ubiquitously in larvae with a *UAS-PEK* inverted repeat (Bloomington Stock Center, TRiP collection) driven by *da-GAL4* (*da > PEK IR*). This approach lead to a reduction of *pek* mRNA to 10.8±0.9% of control as measured by qRT-PCR. We observed that both the *da > PERK IR* larvae showed no increase in polysome levels in either fed or starved conditions compared to control (*da > +*) larvae (Fig. 10C,D).

## DISCUSSION

Protein synthesis has been proposed as a key regulatory process that drives nutrient-dependent growth in *Drosophila*. Our goal in this paper was to investigate the nutrient and signaling requirements for mRNA translation in developing larvae. Our initial ideas were guided by the extensive literature from both tissue culture and *Drosophila* genetics that has identified conserved mechanisms of mRNA translational control (Ma and Blenis, 2009; Proud, 2009; Sonenberg and Hinnebusch, 2009). Drawing on this work, we began with the simple (perhaps simplistic) prediction that amino acids were the key nutritional cue working by activating insulin and TORC1 signaling. However, things were not so straightforward: we found that neither amino acids nor sugars were sufficient to maintain translation, and neither insulin/TORC1 nor eIF2α kinases could fully account for nutrient regulation of mRNA translation. Therefore this work leaves open the question of what the essential nutrient cues and corresponding signaling pathways are that control translation in larvae. Nevertheless, we suggest our findings offer insight into the control of larval translation and suggest further avenues for research in three areas.

### The nature of mRNA translational control in *Drosophila* larvae

We found that mRNA translation was rapidly – within 30 minutes – reduced upon nutrient deprivation. The consistent ribosome profile we observed was a reduction in polysome levels and an increase in free ribosome (40S and 60S) and monosome (80S) levels. This result is consistent with a block in translation initiation. These effects suggest that larvae can rapidly respond to nutrient limitation to suppress the translational control of gene expression. To examine this starvation response further, we used qPCR to analyze the polysomal association of specific mRNAs. We acknowledge that this limited analysis lacked the rigour of genome-wide approaches that have previously been used to explore nutrient and TORC1 translation in yeast, tissue culture, *C. elegans* and adult *Drosophila* (Preiss et al., 2003; Arribere et al., 2011; Hsieh et al., 2012; Thoreen et al., 2012; Morita et al., 2013; Stadler and Fire, 2013; Zid et al., 2009). Nevertheless, our study does provide a picture of translational control in growing larvae. First, we observed that under fully fed conditions for each of the genes we examined, the bulk of their mRNA was associated with large polysomes (>6 ribosomes). This result suggests that a high level of translational control of gene expression drives the rapid and dramatic body growth during the short larval period. Second, we found that upon starvation, the bulk of mRNAs shifted their peak distribution from heavy to lighter polysomes (N = 2–5 ribosomes). This suggests that upon starvation, translation is, in general, reduced but not abolished. With the exception of two ribosomal protein mRNAs we did not see a marked accumulation of mRNAs in the subpolysomal fractions, where one would expect to see untranslated mRNAs (e.g. those associated with mRNPs). Interestingly, two of the genes we tested, FOXO and InR, were previously reported to contain internal ribosome entry sites that were suggested to allow for increased translation under starvation conditions, when normal cap-dependent initiation would be reduced (Marr et al., 2007; Villa-Cuesta et al., 2010). However, we did not observe any evidence of this for these two genes. We also did not observe ‘potentiation’ or ‘homodirectional’ changes in mRNA translation, i.e. mRNAs whose total transcript level were increased by starvation did not show increased translation and vice versa. This type of homodirectional translational control has been described in response to either nutrient changes in *C. elegans* or TOR inhibition in yeast (Preiss et al., 2003; Stadler and Fire, 2013). Instead our findings indicate that the general response is decreased, but not abolished, bulk translation. This effect is probably an important stress survival response to limit excess ATP utilization and unwanted protein synthesis under conditions of nutrient scarcity. Further genome-wide analyses of larval mRNA translation should provide more definitive insight into this potential mechanism.

### What nutrients are required to drive mRNA translation in larvae?

Yeast is the major source of nutrients in almost all laboratory *Drosophila* diets. We found that yeast alone was enough to support mRNA translation when larvae were switched from our complete laboratory diet. A vast literature, mostly in tissue culture, has described how amino acids and sugar can stimulate mRNA translation. However, we found that switching larvae from full food to either amino acids and/or sugar alone was not sufficient to maintain translation. In fact larvae switched to an amino acid/sugar diet for only two hours showed a suppression of mRNA translation similar to that seen with a complete starvation diet, even though these amino acids and sugars were provided at levels comparable to a normal laboratory diet. Over the course of our studies we have used various amounts and sources of sugar and protein (peptone, casamino acids, defined amino acids, serum albumin), with no effect. Hence, we are confident that our negative results are not due to insufficient amounts of sugars and protein. Our results are perhaps surprising given that amino acids are considered as the key regulator of larval growth. Indeed amino acids have been shown to induce endocrine insulin release from the larval brain (Géminard et al., 2009). Moreover, we saw that a sugar + amino acid diet failed to show the strong induction of either *4ebp* or *InR* mRNA normally seen with complete starvation (Fig. 7), suggesting that amino acids and sugars alone can maintain an appreciable level of insulin signaling. Our minimal diets contained metal ions (Magnesium, Sodium, Potassium, Calcium) and salts, however there must be an essential contribution of some other nutrient (not just protein or sugar) present in yeast for full translation. An interesting comparison is with the work of Piper and colleagues. These researchers described a holidic diet (complete with defined macro- and micronutrients, including amino acids and sugar) that could perform as well as a yeast-based diet in allowing optimal lifespan and fecundity in adult *Drosophila* (Piper et al., 2014). However, larvae showed much slower growth and development on this holidic diet compared with a yeast-based food. Moreover, normal larval growth was restored with addition of yeast extract to the holidic diet. This result indicated that some key component other than the complete set of nutrients (such as protein, sugars or cholesterol) were required for optimal larval growth. It is possible that this key component may also limit mRNA translation in our studies. It is interesting to speculate on what this factor may be, and if or how it impinges upon the larval translational machinery.

### What signaling pathways are required to drive mRNA translation in larvae?

In this paper, we also investigated potential signaling pathways that might be important for nutrient-dependent translational control. Our approach was to ask what signaling pathways could maintain translation when animals were switched from full food to starvation diet, i.e. could we identify a signaling pathway(s) sufficient to mediate the effects of nutrition? We chose early starvation periods (2 h and/or 6 hr) when ribosome numbers were not limiting and when (we assumed) gross changes in overall metabolism or physiology would not limit the ability of a candidate signaling pathway to maintain translation. An extensive literature predominantly from cell culture work has described how both insulin and TORC1 signaling regulate mRNA translation, particularly in response to nutrient availability. Hence a prediction when we began our work with was that activation of these pathways would be sufficient to keep translation active following a short-term switch to a starvation diet. However, we found that maintaining high levels of either TORC1 or insulin/PI3K could not prevent the suppression of mRNA translation when larvae were starved. We further examined two effectors of TORC1 – 4EBP and S6K – that have been widely proposed as key regulators mRNA translation. In particular, loss of 4EBP was shown to completely reverse the effect of TORC1 inhibition on mRNA translation and also to promote mammalian cell proliferation (Dowling et al., 2010; Thoreen et al., 2012). However, we observed that neither loss of 4EBP or increased levels of active S6K could maintain translation in starved larvae. Interestingly while *4ebp* null larvae have no growth effects, they are starvation resistant, raising the notion that the predominant role for 4EBP in *Drosophila* is as a ‘brake’ on translation during periods of nutrient deprivation and starvation stress (Teleman et al., 2005; Tettweiler et al., 2005). If so, our data suggest that such a role for 4EBP is not due to a general effect on bulk translation, since translation was not maintained in starved *4ebp* animals. Rather, 4EBP may regulate selective changes in mRNA translation. For example, studies in mammalian cell culture and adult *Drosophila* suggest 4EBP required for selective effects on mitochondrial genes translation (Zid et al., 2009; Morita et al., 2013).

We also examined two eIF2α kinases – GCN2 and PERK. Studies in yeast and cell culture, showed that both kinases are activated under conditions of nutritional stress, including deprivation of amino acids or glucose, to inhibit eIF2α and block translation initiation (Ron and Harding, 2012; Proud, 2014). However, we found that knockdown of either kinase had no effect on starvation-mediated repression of translation. An important caveat is that our work relied on RNAi-mediated knockdown rather than genetic mutants (no published *gcn2* or *pek* mutants exist). However, we did see appreciable reductions in both GCN2 and PEK mRNA using RNAi, and the GCN2 IR was previously used to show a role for GCN2 in mediating effects of amino acid deprivation on larval feeding (Bjordal et al., 2014). Also we saw that the GCN2 knockdown larvae showed a modest increase in translation in fed conditions, suggesting that augmenting eIF2α function may be limiting step in translation in nutrient rich conditions. This notion is consistent with our previous work showing that elevated levels of tRNAiMet – which forms part of ternary complex with eIF2α – can also increase mRNA translation in fed animals (Rideout et al., 2012).

What then are the exact nutrient and signaling requirements for controlling translation in larvae? It is possible that during starvation, the limitation may simply be lack of amino acids as building blocks for protein synthesis, however our results with defined protein diets argue against this. Alternatively, full translation may require a complex combination of nutrients and signaling pathways. Signaling through AMPK has also been proposed as a link between nutrients, particularly glucose, and cellular mRNA translation (Dunlop and Tee, 2013). These effects have often been reported to occur through TORC1, hence our experiments with Rheb overexpression would be predicted to mimic these potential effects of AMPK regulation (Ma and Blenis, 2009). A recent report also showed that AMPK could regulate translation elongation via regulation of eEF2 kinase, in both *C. elegans* and mammalian cells (Leprivier et al., 2013). However, using BLAST searches we found no clear homolog for eEF2 kinase in *Drosophila*, hence it is not clear if this mechanism operates in larvae. Further work is required to define the exact signaling mechanisms that couple nutrients to larval mRNA translation.
